# Abnormal Internal Carotid Artery Kinking in a Cadaver: Morphogenesis and a Potential Link to Alzheimer’s Disease

**DOI:** 10.7759/cureus.91906

**Published:** 2025-09-09

**Authors:** Austin A Charles, Kyle E Thurmann, Alanna C O'Neill, Bailey Wang, Lauren J Suh, Matthew T Culligan, Joshua J Meyer, Mark A Fischione, Manuel E Cevallos

**Affiliations:** 1 Medical Education, Creighton University School of Medicine, Phoenix, USA

**Keywords:** alzheimer's disease, atherosclerosis, carotid artery kinking, cerebrovascular disease, internal carotid artery (ica), vascular dementia, vascular morphogenesis

## Abstract

We report the case of a 95-year-old female cadaver with a documented history of Alzheimer’s disease (AD) and an anteroinferior kinking of the right internal carotid artery (ICA). On dissection, the right ICA bifurcated at the superior one-third of C3 and demonstrated a marked kink with an inferior deviation of 45° followed by superior redirection of 60°, consistent with morphologic criteria for kinking. A digital caliper was used to obtain vessel measurements. The right ICA lumen measured 4.2 mm in diameter with a focal wall thickness of 2.5 mm, exceeding the Mannheim consensus plaque threshold of 1.5 mm and consistent with advanced atherosclerosis. No histologic sections were available to confirm carotid plaque composition, and the diagnosis of AD was based on past medical history without neuropathologic staging. Nevertheless, the coexistence of severe ICA kinking and extensive plaque formation raises the possibility that chronic vascular insufficiency contributed to the decedent's cognitive decline. This interpretation remains speculative, particularly in the context of advanced age and likely coexisting vascular risk factors. This case underscores the importance of recognizing congenital and acquired ICA anomalies during morphologic and clinical evaluation. Quantitative assessment of ICA variation may help refine diagnostic considerations in dementia and support further investigation into the vascular contributions to neurodegeneration.

## Introduction

Internal carotid artery (ICA) kinking is a well-documented vascular anomaly, occurring in 10-25% of the population [1]. In this report, "kinking" refers to a sharp angulation from the expected vessel course, "coiling" to a circular loop, and "tortuosity" to elongated gentle curves. Kinking accounts for 10-31.5% of ICA anomalies, with the METZ 2 (30º-60º) kinking category comprising 10-33% of cases [2]. Its morphogenesis involves the transformation of the aortic arch system [3]. The primitive ICA emerges from the first aortic arch at around 22 days of development (Carnegie stage 11, ~3-mm embryo) [4]. As the first and second aortic arches regress, the third aortic arch becomes the primary contributor to the proximal ICA [3]. The distal ICA originates from the cranial dorsal aorta, with regression of the caudal segment forming its final structure [5].

Kinking of the ICA has been attributed to both developmental and acquired mechanisms. During embryological development, the ICA initially forms with a natural curvature due to its derivation from the third aortic arch and dorsal aorta. Typically, as the fetal heart and great vessels descend into the thoracic cavity, the ICA straightens; however, incomplete descent may result in persistent undulations, loops, or kinks [1,6]. Kinks are also commonly associated with aging due to degenerative changes of the vessel wall [5]. MR angiography studies indicate significant age-related morphological and hemodynamic alterations, with higher prevalence reported among older women [7]. Additional embryologic studies have confirmed the role of pharyngeal arch remodeling in shaping the definitive ICA [8]. Hemodynamic studies using Doppler ultrasound and computational models demonstrate that carotid kinking increases turbulence and pressure drop across the vessel [9,10].

Kinking of the ICA is associated with increased ischemic stroke risk in individuals over 50 and may complicate surgical procedures [11,12]. Blood flow reductions up to 40% at a 60° angle and 60% at a 30° angle have been reported in selected studies [2]. Compared with curving and coiling, kinking is more frequently associated with cerebrovascular disease due to vortex formation [5]. Additionally, observational series suggest that kinking and coiling on angiography in adults produces symptoms of cerebrovascular insufficiency that improve after surgical intervention, although confounding vascular risk factors may contribute. For instance, patients with transient ischemic attacks and angiographic evidence of kinking or coiling of the carotid artery improved following surgery [6].

Vascular dysfunction is increasingly recognized as a contributing factor to Alzheimer’s disease (AD). Reduced cerebral blood flow in AD patients is linked to neurodegeneration, oxidative stress, and inflammation [13,14]. Meta-analytic evidence supports that cerebral hypoperfusion is consistently observed in patients with AD compared to controls [15], reinforcing the vascular hypothesis of neurodegeneration. Here, we report a cadaveric case of unilateral ICA kinking in a donor with a history of AD, with the aim of describing the morphology and discussing how congenital and acquired vascular remodeling may relate to cerebrovascular pathology and dementia.

## Case presentation

**Figure 1:**
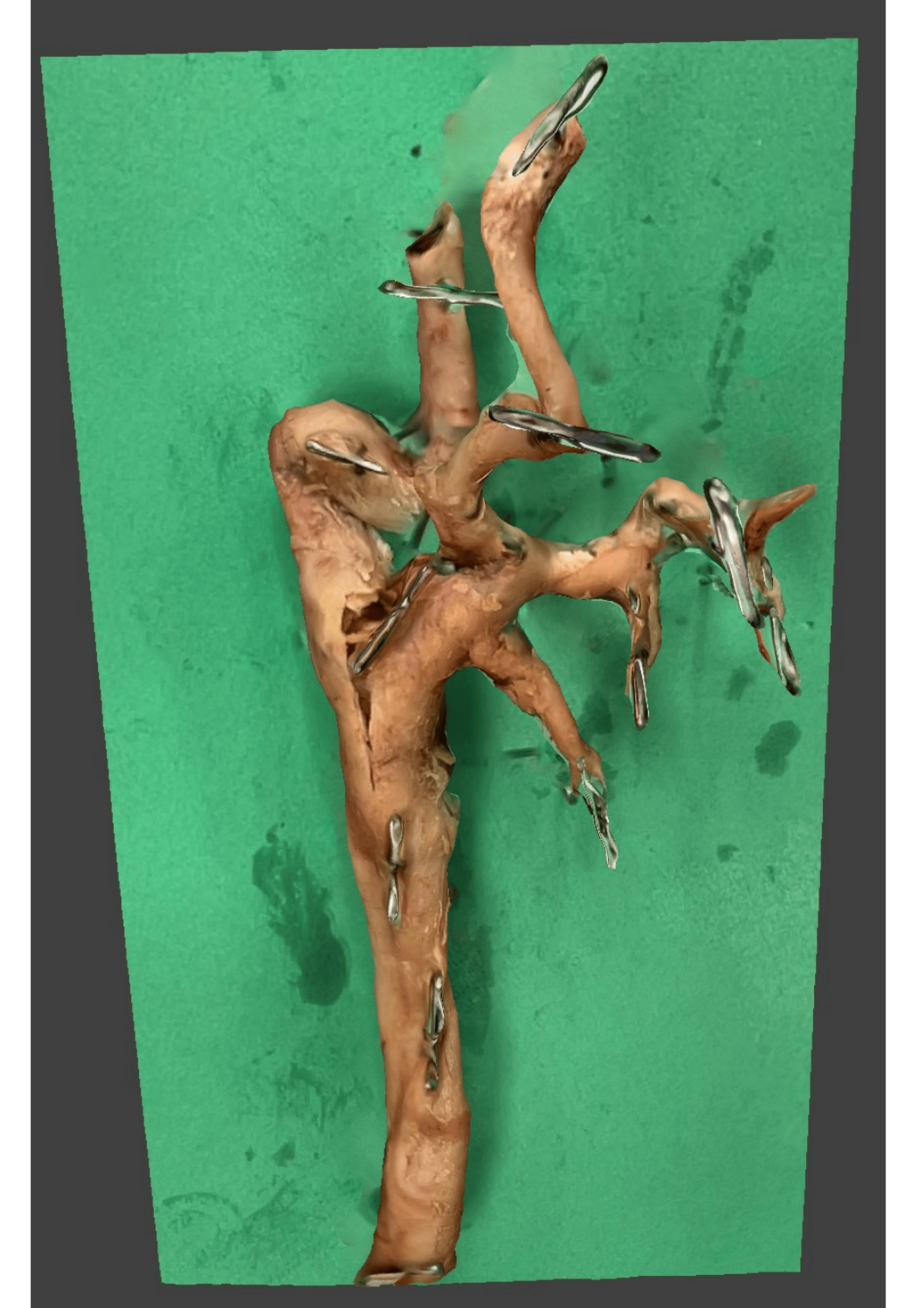
Gross view of the right internal carotid artery kinking The internal carotid artery shows a marked anteroinferior kink just superior to its origin, with the common carotid artery and external carotid artery also visible.

A 95-year-old female cadaver with a history of AD was part of a 30-donor cohort preserved in 3-4% formaldehyde. Cadavers were embalmed by perfusion using standard institutional techniques; the precise interval from death to fixation was not available, and potential fixation-related distortion of vessel caliber cannot be excluded. The authors hereby confirm that every effort was made to comply with all local and international ethical guidelines and laws concerning the use of human cadaveric donors in anatomical research, and de-identified single patient case reports are not required to have IRB approval per Creighton University guidelines. Upon dissection, a pronounced kink was identified in the right ICA, which coursed anteroinferiorly toward the bifurcation of the common carotid artery (CCA) before redirecting superiorly toward the skull, corresponding to a METZ 2 classification (30-60° angulation) (Figure 1).

**Figure 2:**
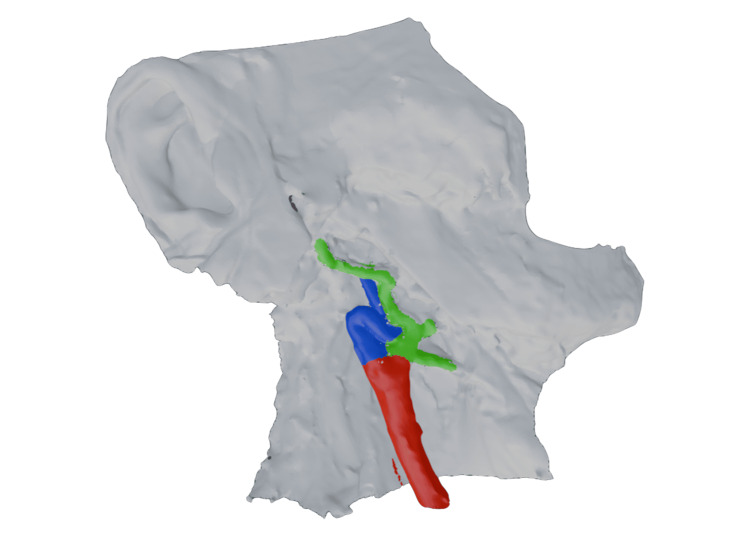
Three-dimensional reconstruction of the right common carotid artery bifurcation Colored 3D model of the right common carotid artery bifurcation, highlighting the severity of the kink and its spatial relationship. Red indicates common carotid artery, blue indicates internal carotid artery, and green indicates external carotid artery

**Figure 3:**
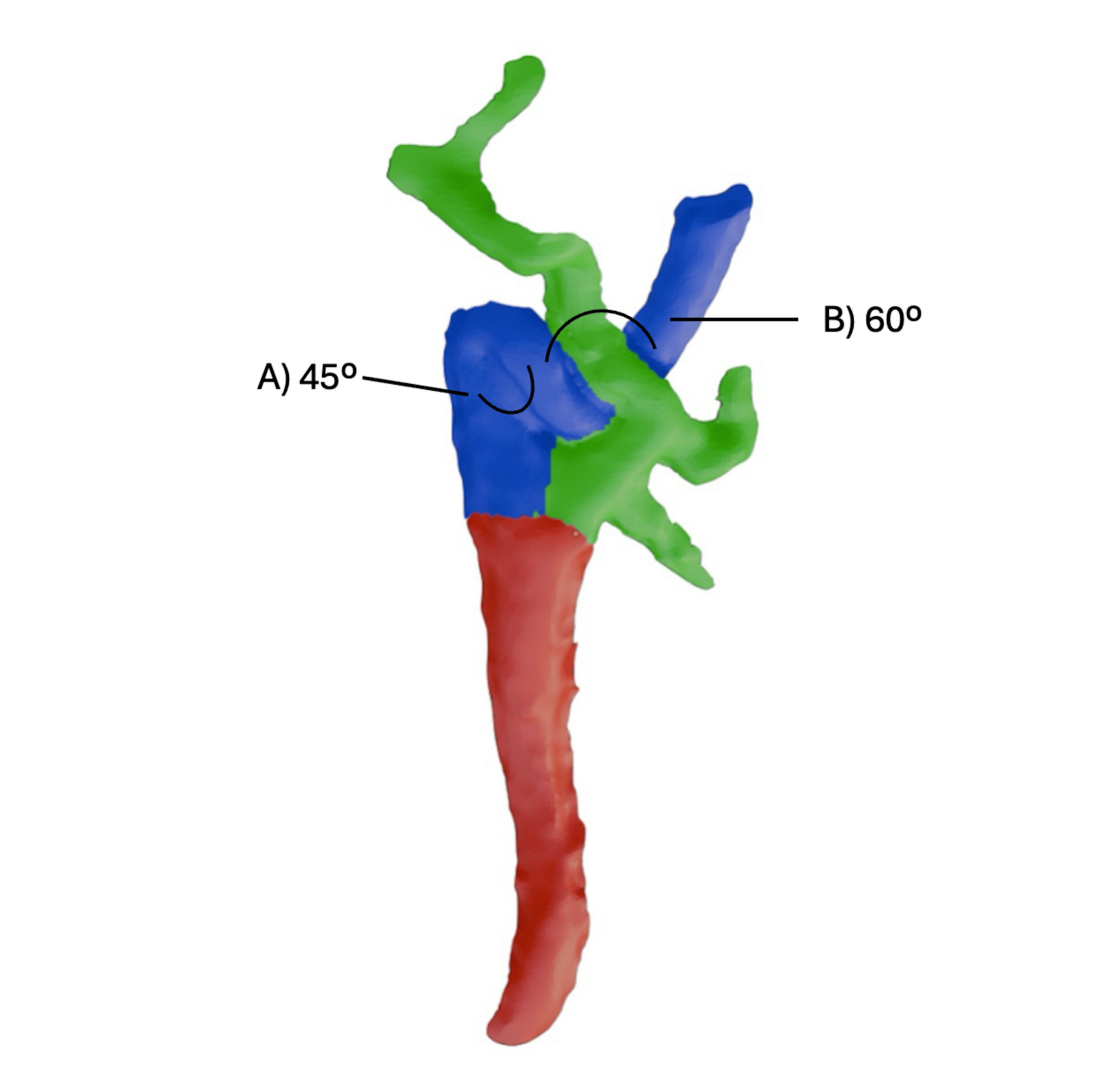
Measurement of the kinking angles of the internal carotid artery Colored 3D model depicting how kinking angles of the internal carotid artery were measured. The initial anteroinferior kink (A) measured 45º while the superior redirection (B) measured 60º. Red indicates common carotid artery, blue indicates internal carotid artery, and green indicates external carotid artery

The anomaly was documented using 3D scanning (Figure 2). The left ICA appeared unremarkable in its anatomical presentation. The ICA kink exhibited angles of 45° and 60°, extending 15.9 mm before redirecting toward the bifurcation (Figure 3). The mean right CCA wall thickness was 2.5 mm, with visible calcification at the bifurcation. No histologic sections were available to further characterize plaque composition. The right CCA bifurcated at the superior one-third of C3, whereas the left CCA bifurcated at the superior one-third of C4. The right ICA diameter was 2.4 mm at the middle cerebral artery level, compared to 3.1 mm on the left. No calcification was observed contralaterally. Percent stenosis could not be calculated due to the absence of pre-mortem angiography. All measurements were obtained using a digital caliper and protractor.

A board-certified pathologist evaluated the brain and vasculature. Examination revealed cortical atrophy and significant gyral thinning, with narrowing of the gyri and widening of the sulci, affecting multiple lobes. Extensive plaque formation was found in the right CCA, extending into the ICA and external carotid artery, as well as in the ICA at the level of the middle cerebral artery and posterior communicating artery. Neuropathological staging was not available, and the diagnosis of AD was based on documented medical history. Additional donor comorbidities and vascular risk factors were not available. This was the only donor in the 30-cadaver cohort to demonstrate ICA kinking, corresponding to a prevalence of 3.3% based on this population.

## Discussion

AD is a multifactorial condition with a possible vascular-related etiology. The vascular hypothesis of AD proposes that chronic cerebral hypoperfusion contributes to neurodegenerative changes, including the development of beta-amyloid plaques and neurofibrillary tangles in the brain [16]. In this case, the right ICA kinking likely contributed to hemodynamic alterations, which may have exacerbated cerebrovascular dysfunction and led to extensive atherosclerotic plaque formation. A carotid wall thickness of 2.5 mm is abnormally high and exceeds thresholds commonly used to define plaque (e.g., Mannheim consensus >1.5 mm), consistent with advanced atherosclerosis [17,18]. The presence of extensive plaque suggests that ischemia and infarcts, rather than amyloid-driven mechanisms, played a primary role in the decedent’s cognitive decline. Aligning with the vascular hypothesis, the pathologist suggested that the diagnosis of AD may have been due to multi-infarct dementia (MID) rather than the typical amyloid plaque-driven pathogenesis of AD due to the severity of plaque formation. The severity of plaque formation likely resulted in MID, which mimicked AD clinically and contributed to its diagnosis. This potential overlap between MID and AD clinical presentations, particularly in the context of significant cerebrovascular pathology, has been previously documented in neuropathological studies of mixed dementia syndromes [19].

While causality cannot be established, the markedly kinked ICA with significant plaque formation raises concerns about whether chronic hypoperfusion accelerates neurodegeneration. Chronic cerebral hypoperfusion has been increasingly recognized as a causative contributor to neurodegnerative changes in both animal models and human studies, supporting this hypothesis [20]. Given that ICA kinking has been associated with embryologic variations in vascular regression and remodeling, individuals with congenital ICA anomalies may have an increased risk for hemodynamic disturbances and cerebrovascular disease over time [5,10]. The extensive plaque formation from the kinked ICA may have contributed to MID, culminating in a clinical diagnosis of AD. This highlights the critical role of vascular morphogenesis and hemodynamic alterations in brain degeneration. Midlife vascular risk factors, including hypertension and hypercholesterolemia, have been associated with elevated incidence of dementia, further implicating vascular burden as a key modifiable contributor [21].

The overlap in symptomatology between MID and AD underscores the need for further research into vascular contributions to neurodegeneration. Identifying ICA anomalies and monitoring atherosclerotic progression in living patients could provide valuable insight into long-term neurodegenerative risk, particularly in individuals with other cerebrovascular risk factors such as hypertension and hypercholesterolemia. Because dementia significantly impacts quality of life, future research should explore the distinction between vascular and amyloid-driven pathways in neurodegeneration, which may ultimately refine diagnostic and therapeutic approaches.

## Conclusions

This case highlights the potential role of ICA kinking in contributing to chronic cerebral hypoperfusion and extensive atherosclerotic plaque formation, which may exacerbate or mimic the clinical presentation of AD. Recognition of vascular anomalies such as ICA kinking is important for clinicians, as these findings may underlie neurodegenerative symptoms typically attributed to amyloid pathology. Distinguishing between vascular-driven MID and classic AD has significant implications for diagnosis, prognosis, and management. To our knowledge, prior cadaveric case reports describe ICA kinking/variation without donor-level neurodegenerative diagnoses, and clinical case reports of ICA kinking with cognitive symptoms do not provide AD neuropathology. We therefore believe that this is among the first cadaveric case reports to describe severe ICA kinking in a donor with a documented history of AD, situating a focal vascular anomaly within the broader context of dementia research. Further research is warranted to clarify the relationship between developmental vascular anomalies, long-term cerebrovascular health, and neurodegenerative disease.
